# Greed: What Is It Good for?

**DOI:** 10.1177/01461672221140355

**Published:** 2022-12-28

**Authors:** Karlijn Hoyer, Marcel Zeelenberg, Seger M. Breugelmans

**Affiliations:** 1 Tilburg University, The Netherlands; 2 Vrije Universiteit Amsterdam, The Netherlands

**Keywords:** greed, self-interest, economic outcomes, evolutionary outcomes, well-being

## Abstract

What is greed good for? Greed is ubiquitous, suggesting that it must have some benefits, but it is also often condemned. In a representative sample of the Dutch population (*N* = 2,367, 51.3% female, *M*_age_ = 54.06, *SD* = 17.90), we examined two questions. First, inspired by Eriksson et al., we studied whether greedy people generate more personal and household income (economic outcomes), have more sexual partners, longer relationships, and more offspring (evolutionary outcomes), and are more satisfied in life (psychological outcomes). We found that greedy individuals had higher economic outcomes, mixed evolutionary outcomes, and lower psychological outcomes. Second, we compared greed and self-interest. We found that they differed in terms of economic outcomes, and partly in terms of evolutionary outcomes, but that they were similar in terms of psychological outcomes. This research provides insights into what greed is and does. Directions for further research are discussed.

Greed is as intriguing as it is controversial. Across cultures and historical periods, greed is considered an important motive and almost equally often has greed been condemned as being immoral, sinful, or outright evil (e.g., Haynes, 2021; Tickle, 2004). Most people do not want to be called greedy, as it has a negative connotation (Gilliland & Anderson, 2011). Are there benefits to being greedy, despite its condemnation and suppression? In other words, is there anything that greed is good for?

We approach this question from a dispositional angle. There are individual differences in how greedy people are, and these differences appear to be normally distributed in the population (Preston & Vickers, 2014; Zeelenberg & Breugelmans, 2022). There are various valid and reliable instruments to assess the greediness of people (for a comparison, see Zeelenberg et al., 2022). The availability of these instruments allows us to test how greed is related to various life outcomes and thus may help us to answer the question what greed is good for.

This article presents a study among a representative sample of the Dutch population (*N* = 2,367) to test how dispositional greed relates to economic, evolutionary, and psychological outcomes, building on a study by Eriksson et al. (2020) who studied the economic and evolutionary correlates of self-interest. Since greed and self-interest are related, we also include a comparison between these constructs in our analyses. Before turning to the studies, let us explain our approach to greed, its relationship with self-interest, and the predictions for the various types of outcomes.

## Greed as Good or Bad

A returning question in the literature on greed is whether greed is good or bad (Oka & Kuijt, 2014; Verburg, 2012; Zeelenberg & Breugelmans, 2022). The case for greed being bad is most frequently encountered: Greed is excessive, wasteful, resulting in accumulation beyond what is needed, and often harmful to other people (Balot, 2001; Gilliland & Anderson, 2011; Helzer & Rosenzweig, 2020; Lambie & Stickl Haugen, 2019). Indeed, research has shown greed to be related to dishonest and harmful behaviors (Li et al., 2021; Seuntjens et al., 2019) and to the dark triad of personality (i.e., Machiavellianism, narcissism, and psychopathy; Sekhar et al., 2020). Greed has also been blamed for causing the 2008 financial crisis (Hoyer, Zeisberger, et al., 2021). This may be one of the reasons why all major religions condemn greed as being immoral, sinful, or evil (Bloch, 1984; Tickle, 2004). In sum, there is a clear case to be made for views of greed being bad.

Views of greed being good may be less salient in the psychological literature, but they are by no means less important (Zeelenberg & Breugelmans, 2022). In economics, for example, greed is argued to stimulate productivity and economic growth (Bruhn & Lowrey, 2012; Greenfeld, 2001) and to motivate the development of new products and industries, which in turn increases employment, wealth, and well-being (Melleuish, 2009; Wight, 2005). Similar arguments have been made by evolutionary theorists, such as “the acquisitive nature of mankind is absolutely central to his prospects for survival.” (Jett, 2000, p. 11). Greed is argued to be essential for human welfare (Williams, 2000) and to facilitate self-preservation because greedy behaviors (e.g., hoarding) provide an evolutionary advantage for those living in scarce environments (Cassill & Watkins, 2004; Robertson, 2001). Thus, there is also a case to be made for greed being good.

It is important to note that both views of greed being bad and greed being good are mostly based on the effects greed has on other people or on society as a whole. The question as to whether greed is (dis)advantageous for greedy individuals themselves is seldom addressed. We believe this question is relevant. Are there differences in life outcomes between greedy and less greedy individuals? In the present research, inspired by the work of Eriksson et al. (2020), we address potential benefits referring to economic outcomes, evolutionary outcomes, and psychological outcomes. Before venturing into these types of outcomes, we first explain what greed is and how it can be measured.

## Greed

Research on the dynamics of greed has increased substantially over the last decade, catalyzed by Wang and Murnighan’s (2011) comprehensive review of ideas from economics, politics, philosophy, history, game theory, and psychology. They concluded that despite the long intellectual history of greed, empirical greed research was rare, probably due to the lack of consensus on how to define greed.

The recent increase in research led to consensus on the desire to acquire more being a defining feature of greed, often referred to as an *excessive* desire (e.g., Mussel et al., 2015; Wang et al., 2011) or an *insatiable* desire (e.g., Seuntjens, Zeelenberg, Breugelmans, & Van de Ven, 2015). Differences between definitions can be found in whether additional features are also seen as part of greed. For example, there is still discussion on the extent to which greed generalizes beyond material goods, on whether greed includes harm-to-others, and on whether greed also includes a retention motive (e.g., Lambie & Stickl Haugen, 2019; Mussel et al., 2018; Zeelenberg et al., 2022).

Greed is clearly related to materialism, maximization, envy, and self-interest (e.g., Krekels, 2015; Seuntjens, Zeelenberg, Van de Ven, & Breugelmans, 2015), as all reflect wanting more. Materialism entails a desire for material possessions to signal success in life (Richins, 2004). Greed is not only felt for outcomes that signal success or status but can also be experienced for nonmaterial outcomes such as sex, food, or power (Seuntjens, Zeelenberg, Van de Ven, & Breugelmans, 2015). The latter makes greed a broader construct.

For maximizers, the ultimate goal is to acquire the best outcome (Schwartz et al., 2002). Greedy people, on the contrary, simply desire to acquire more and a greedy person might even go into debt to buy the desired product (Livingstone & Lunt, 1992). Greed is thus not necessarily a rational endeavor (TerBush, 2021), while maximization, per definition, involves rational balancing of costs and benefits (Seuntjens, Zeelenberg, Van de Ven, & Breugelmans, 2015).

Envy is felt when others are better off while we do not feel they deserve it (Van de Ven et al., 2011). Greed, in contrast, can be unrelated to what others have. “Envy is thus driven by an external factor (wanting what others have) whereas greed is driven by internal motivations (wanting more)” (Seuntjens, Zeelenberg, Van de Ven, & Breugelmans, 2015, p. 4). The two constructs frequently co-occur. For example, Crusius and Lange (2021) found that greedy people also experience more envy.

Most important for our current discussion is self-interest, which we will compare with greed in more detail later on. We define self-interest as “a concern for one’s own advantage and well-being” (Merriam-Webster Online Dictionary, n.d.-b). Self-interested individuals care about their own outcomes. Greedy individuals are similar, but mostly motivated by the insatiable desire to get more; more than is needed and sometimes even more than is possible. This constant striving for more and dissatisfaction with what they have, means that greedy individuals may act in ways that are not rational (Seuntjens, Zeelenberg, Van de Ven, & Breugelmans, 2015; TerBush, 2021).

In the last decade, various self-report scales have been published: the Greed Avoidance subscale from the HEXACO (Lee & Ashton, 2004), the Greed-subscale from the Vices and Virtues Scales (Veselka et al., 2014), the Greed Trait Measure (Mussel et al., 2015), two Dispositional Greed Scales (DGSs; Krekels & Pandelaere, 2015; Seuntjens, Zeelenberg, Van de Ven, & Breugelmans, 2015), the GR€€D scale (Mussel & Hewig, 2016), and the Multidimensional Dispositional Greed Assessment (MDGA; Lambie et al., 2022).

Mussel et al. (2018) investigated the convergent validity of the five scales developed between 2014 and 2016 and found that “despite the conceptual differences, these scales converged on a common latent factor” (p. 249). All scales also correlated with greed-related behavior. Zeelenberg et al. (2022) compared the same scales and concluded that “all scales can be used to assess dispositional greed, as all the scales are reliable and correlate highly” (p. 98). Thus, there is strong convergence in measures of dispositional greed, which all include items relating to the insatiable desire for more, which is central in definitions of greed given by most researchers.

In our research, we use the DGS (Seuntjens, Zeelenberg, Van de Ven, & Breugelmans, 2015), which is most commonly used. It is reliable, valid, stable over time, and predictive of a wide variety of relevant social, economic, and financial behaviors. The DGS has been translated and validated for use in various countries, such as Brazil (Freires et al., 2019), China (Liu, Sun, Ding, et al., 2019), Japan (Masui et al., 2018), Belarus (Fourmanov & Shirko, 2020), and Russia (Poluektova et al., 2022).

## Important Life Outcomes

In this article, we translate the question as to what greed is good for to the question whether dispositional greed relates positively to various important life outcomes. This approach was inspired by Eriksson et al. (2020), who studied how self-interest relates to economic and evolutionary outcomes (i.e., number of offspring and income). They noted that theories in economics and evolutionary biology emphasize the power of self-interest but that clear empirical support for such views in the psychological literature was lacking.

Drawing on a large number of community sample data sets, Eriksson et al. (2020) constructed indexes of self-interest from the variables available in the data. They “define selfish motivation as the inverse of (prosocial or) otherish motivation: wanting or striving to benefit the self without regard for the well-being of others” (p. 532). Accordingly, in their first study, self-interest was measured with a 3-item Prosocial Motivation scale, with self-interest being indicated by a low score on this scale.^1^ They found that self-interested individuals had a lower personal income as well as a lower household income than prosocial individuals^2^ and that they also had fewer children. They concluded that self-interested individuals are economically and evolutionary worse-off than prosocial individuals.

We think these findings are also relevant for the question as to what greed is good for. Although not identical, self-interest and greed are clearly related constructs. Nevertheless, we expected greed to have different relationships with life outcomes than self-interest. To explain these expectations, we first explain which life outcomes we employed and what the similarities and differences between greed and self-interest are.

We deemed a combination of objective indicators of evolutionary and economic life outcomes and subjective indicators of psychological life outcomes to provide the best answer to the question what greed is good for. These indicators include those used by Eriksson et al. (2020), income and offspring. For evolutionary outcomes, we included additional indicators of life history strategies (MacArthur & Wilson, 1967): number of sexual partners and the duration of the longest romantic relationship. Having multiple sexual partners increases the chance of having more and more genetically diverse offspring while investing fewer resources in each (an *r*-strategy); having longer romantic relationships limits the number of offspring but invests more resources in each, increasing their probability of surviving to adulthood (a K-strategy). For subjective, psychological outcomes, we added the “Satisfaction-with-Life” scale (Diener et al., 1985), which is the most widely used and validated indicator of subjective psychological well-being. Together, these indicators should allow us to address what greed is good for, but also to what extent greed differs from self-interest.

Note that in this article, we prefer to use the term life outcomes rather than success (the term used by Eriksson et al., 2020). We do this because for various outcomes, it is difficult to unequivocally judge what is actually good or bad for individuals themselves, whether more of it is actually better or whether this is a valued characteristics by society. It could be argued that having more offspring or sexual partners means an individual is successful, and according to evolutionary reasoning this would be the case. But others might have good reasons to have fewer offspring and sexual partners and be happy with these outcomes. They might, for example, choose not to have children because they fear that doing so will amplify global warming (one fewer child could save approximately 58.6 metric tons of carbon emissions each year in developed countries; Wynes & Nicholas, 2017), or because they have life goals that are harder to achieve with children. Similarly, having more money is not good per se (even though economic theory would argue it cannot be bad). The marginal increase of happiness as a consequence of more income quickly diminishes and time acquiring more income may well be better spent on other activities when maximizing well-being (Kahneman & Deaton, 2010). The case for success is perhaps most easily made for life satisfaction as a proxy for psychological success. However, there are stable differences in people’s baseline happiness as well as many situational factors affecting life satisfaction (Diener & Lucas, 1999), so also for this indicator, we prefer to refer to life outcomes.

## Greed and Self-Interest

Greed and self-interest are intrinsically related. Both constructs refer to a desire to obtain outcomes for oneself. For example, a leading dictionary describes greed as the “*selfish* and excessive desire for more of something (as money) than is needed” (Merriam-Webster Online Dictionary, n.d.-a, italics added). Some even argued that greed should be seen as “*a selfish motivation to acquire an unfairly excessive amount of a resource, at the expense of others*.” (Cardella et al., 2019, p. 580, italics in the original). A prototype analysis of greed indeed found that self-interest was among the most frequently mentioned features of greed (Seuntjens, Zeelenberg, Breugelmans, & Van de Ven, 2015).

Adam Smith (1759, 1776) is well-known for praising the benefits of self-interest, but he may be less well-known for having a very negative opinion about greed (which he refers to as avarice), expressed both in the “*Theory of Moral Sentiments*” and in the “*Wealth of Nations*.” In the latter, A. Smith (1776, p. 305) wrote: “Avarice and injustice are always short-sighted” and discussed many examples of how greedy behavior would hurt the wealth of businessmen. According to Smith, greed does not equal self-interest because greed disregards the welfare of others and could even work against the self-interest of people. In subsequent work, both in economics and in psychology, greed and self-interested have been treated as distinct constructs.

The assumption of self-interest in standard economic theory refers to focusing only on one’s own outcomes without taking the outcome of others into account (e.g., Luce & Raiffa, 1957; Von Neuman & Morgenstern, 1947). Similarly in psychological theory, self-interest refers to a stronger weighting of own outcomes than of other people’s outcomes (e.g., Van Lange et al., 1997). In contrast, the axiom of greed in economic theory refers to agents always preferring more of a desirable good compared to less (Lea et al., 1987). Similarly, in psychological terms, greed has been defined as the “dissatisfaction of not having enough, combined with the desire to acquire more” (Seuntjens, Zeelenberg, Van de Ven, & Breugelmans, 2015, p. 928). Thus, while self-interest is defined in terms of a comparison between self and others, greed is about acquisitiveness and does not necessarily involve other people.

While self-interest in economics is considered to be rational (i.e., leading to maximal utility for the individual), greed is not necessarily rational (TerBush, 2021). For example, Zeelenberg et al. (2020) found that greedy people worked harder, at the expense of enjoying leisure time, even when the outcomes of their work could not be consumed.

Self-interest is most often assessed in terms of people’s Social Value Orientation (SVO; Murphy et al., 2011; Van Lange et al., 1997). The SVO framework assumes that “people vary in their motivations or goals when evaluating different source allocations between themselves and another person” (Murphy et al., 2011, p. 771). Seuntjens, Zeelenberg, Van de Ven, and Breugelmans (2015) found in two different samples (*N*_1_ = 167, *N*_2_ = 236) that self-interest (measured with two different SVO measures) and greed (measured with the DGS) correlated. But these correlations were low, between .12 (Van Lange et al. measure) and .21 (Murphy et al. measure). The pattern of correlations, with a large number of other constructs that are theoretically relevant, displayed in their Table 3, provides insight into the nomological network of greed. We reanalyzed the data of Seuntjens et al. and also constructed, in a similar manner, the nomological network of self-interest (with both the Van Lange et al. and the Murphy et al. measure). Results can be found in Table 1. There are a number of differences between the nomological network of greed and self-interest that we would like to highlight here. In the data of Seuntjens et al., greed is related to more maximization, more envy, less self-control, more impulsiveness, and less satisfaction with life, while self-interest is not related to either of these constructs. Also, greed is related to less emotional stability, while self-interest is related to more emotional stability. These differences make sense theoretically because greed is, as explained, not necessarily a rational pursuit, while self-interest generally is. These differences are also a first clear empirical indication of the distinctiveness of greed and self-interest. The reanalysis of the data of Seuntjens et al. also shows that both greed and self-interest are related to more materialism, more psychological entitlement, more psychopathy, less perspective taking, and less emphatic concern, which also makes sense theoretically because both greedy and self-interested people focus on the fulfillment of their own needs. Consequently, with regard to this study, we expected greed and self-interest to have different relationships with various economic, evolutionary, and psychological outcomes (see top of Table 2).

**Table 1.** Correlations of the Dispositional Greed Scale and Two Measures of Self-interest (SVO) With Other Measures Using Data From Seuntjens, Zeelenberg, Van de Ven, and Breugelmans (2015) Study 1.

Constructs	Sample 1 (*N* = 167)		Sample 2 (*N* = 236)		
	Greed	SVO (Van Lange et al., 1997)	Greed	SVO (Van Lange et al., 1997)	SVO (Murphy et al., 2011)
Social value orientation (Van Lange et al., 1997)	.21**		.17**		
Social value orientation (Murphy et al., 2011)			.12	.52***	
Maximization scale (Nenkov et al., 2008)	.29***	–.07	.25***	.06	.02
Dispositional envy scale (R. H. Smith et al., 1999)	.34***	.15	.33**	.08	–.07
Material values scale (Richins, 2004; Richins & Dawson, 1992)	.55***	.23**	.64***	.21**	.19**
Self-control scale (Tangney et al., 2004)	–.26**	–.13	–.22**	–.01	.09
Impulsiveness (Eysenck et al., 1985)	.24**	.11			
Temporal preferences (Mahajan & Tarozzi, 2012)			–.08	.00	.03
Risk aversion (Holt & Laury, 2002)			.04	–.06	–.02
Psychological entitlement scale (Campbell et al., 2004)	.33***	.25**			
Self-report psychopathy scale (Williams et al., 2003)	.32***	.30***	.22**	.28***	.27***
Perspective taking—interpersonal reactivity index (Davis, 1980)	–.33***	–.30***			
Emphatic concern—interpersonal reactivity index (Davis, 1980)	–.21**	–.21**			
Rosenberg self-esteem scale (Rosenberg, 1965)			–.20**	.09	.13*
Satisfaction with life scale (Diener et al., 1985)			–.17**	.02	.01
Beck depression inventory (Beck, 1967)			.10	–.04	–.03
Iowa–Netherlands comparison orientation measure (Gibbons & Buunk, 1999)	.11	.07			
Extraversion (TIPI, Gosling et al., 2003; IPIP, Goldberg, 1992)	–.03	.09	.03	.09	.04
Agreeableness: TIPI, (Gosling et al., 2003), IPIP (Goldberg, 1992)	–.11	–.01	–.12	–.16*	–.12
Conscientiousness: TIPI (Gosling et al., 2003), IPIP (Goldberg, 1992)	–.12	–.12	–.08	–.03	.00
Emotional stability: TIPI (Gosling et al., 2003), IPIP (Goldberg, 1992)	–.17*	–.00	–.14*	.16*	.13*
Openness: TIPI (Gosling et al., 2003), IPIP (Goldberg, 1992)	–.22**	–.06	–.09	.04	.02

*Note.* Greed refers to the Dispositional Greed Scale (Seuntjens, Zeelenberg, Van de Ven, & Breugelmans, 2015). SVO refers to the Social Value Orientation measure, all coded such that higher scores indicated more self-interest. When correlations are not reported, the scale was not measured in that sample. SVO = Social Value Orientation; TIPI = Ten Item Personality Inventory; IPIP = International Personality Item Pool.

* *p* < .05. **p < .01. ***p < .001.

**Table 2.** Summary of Hypothesized (Top) and Empirical (Bottom) Relations Between Greed and Self-Interest and Life Outcomes.

	Measure	Life outcomes					
		Economic		Evolutionary			Psychological
Relations		Personal income	Household income	Biological children	Longest relationship	Sexual partners	Satisfaction with life
Hypothesized relations	Greed	↑	↑	↓	↓	↑	↓
	Self-interest	↓	↓	↓	↓	↓	↓
Empirical relations	Greed	○	↑	↓	↓	↑	↓
	Self-interest (PM)	○	○	↓	↓	○	○
	Self-interest (SVO)	○	↓	○	↑	○	↓

*Note.* For the hypothesized relations, ↑ indicates an expected positive relation, while ↓ an expected negative relation; For the empirical relations, ↑ indicates a significant (*p* < .05), positive relation; ↓ indicates a significant, negative relation; ○ indicates no significant relation. Greed refers to the Dispositional Greed Scale (Seuntjens, Zeelenberg, Van de Ven, & Breugelmans, 2015), PM refers to the prosocial motivation measure (Eriksson et al., 2020) and SVO refers to the Social Value Orientation measure (Murphy et al., 2011), all variables are coded such that higher scores indicated a more greed and a more self-interested orientation. PM = prosocial motivation; SVO = social value orientation measure.

## Hypotheses

With regard to *economic outcomes*, we expected a positive relationship between greed and income, although current findings are mixed. Seuntjens, Zeelenberg, Van de Ven, and Breugelmans (2015) found no relationship in a sample of adults, but Seuntjens et al. (2016) found greed to be associated with higher income in a sample of adolescents. Van Muijen and Melse (2015) found no relationship for younger respondents (i.e., younger than 36) but did find a negative relationship for older respondents (i.e., older than 35). Interestingly, for specific occupations such as sales managers, they found a positive relationship between greed and income. Greedy sales managers earned on average 10% more than middle-income (i.e., about €3,000). At the same time, they found that respondents with an above-average greed score mainly worked in fields such as sales and finance. Following the reasoning of economists that greed is good for economic growth and that greed has been empirically linked to overearning (Zeelenberg et al., 2020), we hypothesized that greed is positively related to income (both personal and household income).

We also expected to replicate Eriksson et al.’s (2020) finding that self-interest is negatively related to personal and household income. Eriksson et al. had various reason to expect this relationship. First, prosocials are granted more positions of status (e.g., Hardy & Van Vugt, 2006), which are generally accompanied by larger material outcomes. Second, many higher paying jobs require the ability to work well with others, something prosocials are better at than self-interested individuals (e.g., Van Doesum et al., 2013). Finally, social contacts are key sources of information about employment opportunities, and prosocials are better at building relationships than self-interested individuals (Crocker et al., 2017).

With regard to *evolutionary outcomes*, we expected that greedy individuals have fewer children and shorter relationships, but more sexual partners. These expectations follow from two possibly opposing effects of greed. On one hand, the drive to always want more that is typical for greed, might also be reflected in a drive to want more children. In addition, previous research has shown that the desire to acquire more, which is core to the definition of greed, also manifests itself in a desire for as many casual sexual partners as possible (Seuntjens, Zeelenberg, Van de Ven, & Breugelmans, 2015). This desire might be so strong that it motivates greedy individuals to (temporarily) invest more in their social relationships to attain this goal. Furthermore, greedy individuals indicated a stronger desire to cheat on their partner and were more easily lured into this behavior (Seuntjens et al., 2019). However, on the other hand, whether greedy individuals actually manage to reach these goals depends on the willingness of other people to engage with them. Being greedy is not generally looked well upon and greed correlates with a variety of negative personality traits, like egoism and meanness (Mussel & Hewig, 2016; Seuntjens, Zeelenberg, Van de Ven, & Breugelmans, 2015). Thus, we expected both greed and self-interest to be negatively related to long-term indicators of evolutionary outcomes, namely the number of biological children and the length of the longest romantic relationship, but positively to a more short-term indicator, number of sexual partners.

Furthermore, we expected to replicate Eriksson et al. (2020) who found that self-interested individuals had fewer children than prosocial individuals. They give two potential reasons for this negative relationship. First, prosociality is related to having better relationships (Crocker et al., 2017), and this creates more opportunities to have children. Second, self-interested individuals might be less motivated to have children, because having children generally requires personal sacrifices.

With regard to *psychological outcomes*, greed has been found to be related to lower psychological well-being because it leads to more emotional instability, lower self-esteem, and lower satisfaction with life (Krekels & Pandelaere, 2015; Masui et al., 2018; Poluektova et al., 2022; Seuntjens, Zeelenberg, Van de Ven, & Breugelmans, 2015; Zeelenberg et al., 2020). Hence, we expected to find that both greed and self-interest are related to lower psychological well-being. A recent review revealed that a prosocial motivation is positively related to psychological well-being, physical health, and social relationships (Crocker et al., 2017). This implies that being self-interested would have negative consequences for psychological outcomes.

The above predictions, and the accompanying findings, can be found in Table 2. This study was preregistered^3^ via AsPredicted/22694, https://aspredicted.org/zt27s.pdf. Data were collected in May 2019. Processed data^4^ and code can be found on https://researchbox.org/572.

## Method

### Participants

Participants (*N* = 2,367, 51.3% female, age range 16–95, *M*_age_ = 54.06, *SD* = 17.90) were members of the Dutch nationally representative LISS (Longitudinal internet Studies for the Social Sciences) panel.^5^ Following the preregistration, we excluded 50 participants who did not complete all key scales (i.e., the DGS, Prosocial Motivation scale and SVO-slider). *N* varies across the life outcomes, depending on the responses. Roughly half filled out the DGS at the beginning of the survey, the others at the end.^6^ The survey was administered in Dutch.

### Measures

In addition to the administered scales, we included a number of demographic measures that are available for the LISS panel members (such as income, age, and gender). Individual differences in greed were measured with the 7-item DGS (Seuntjens, Zeelenberg, Van de Ven, & Breugelmans, 2015). Example items are: “I always want more” and “It doesn’t matter how much I have. I’m never completely satisfied” (1 = *Strongly disagree*, to 5 = *Strongly agree*).

Self-interest was assessed with two different scales measuring individual differences in how much people care about themselves and others. The first was the 3-item Prosocial Motivation scale of Eriksson et al. (2020). The items were: “People should be willing to help others who are less fortunate,” “Personally assisting people in trouble is very important to me,” and “These days people need to look after themselves and not overly worry about others” (rated 1 = *Strongly disagree* to 4 = *Strongly agree*). For the present purposes, this was coded such that a higher score reflected more self-interest (and thus a lower prosocial motivation). The second was the more commonly used SVO-slider (Murphy et al., 2011), which comprises of six decomposed games. An SVO score was computed for each subject following the procedure specified by Murphy et al. For the present purposes, this was coded such that a higher score reflected more self-interest (i.e., a more proself and less prosocial orientation).

Economic outcomes were assessed by two gross monthly income variables that were both available from the LISS panel: personal^7^ and household income, both in Euros and imputed.^8^ These were open questions in which participants could specify their personal gross monthly income and gross household income of all household members combined in euros. Data from two extreme outliers with personal monthly incomes above €440,000, while not being (self)employed, were excluded from the analysis.

Evolutionary outcomes were assessed by three questions. The first question was adopted from Eriksson et al. (2020) and asked about the number of biological children (including deceased children). The second question asked about the length of the longest romantic relationship in years and months (data from six participants were excluded because they indicated a relationship length greater than their age). The third question asked about the number of sexual partners during their lifetime.

Psychological outcomes were assessed by the 5-item Satisfaction-with-Life scale (SWLS, Diener et al., 1985). Example items are: “In most ways my life is close to ideal” and “I am satisfied with my life” (rated 1 = *Strongly disagree*, to 7 = *Strongly agree*).

## Results

The results are summarized in the bottom panel of Table 2. Table 3 shows the means, standard deviations, and scale reliabilities of all relevant variables, and the correlations of greed and the two self-interest measures (that were positively correlated, *r* = .23, *p* < .001). The full correlation Table can be found in Supplemental Appendix 1. Dispositional greed correlated positively with both measures of self-interest. The greedier people were, the more self-interested they were.

**Table 3.** Means, Standard Deviations, of All Measured Constructs and Correlations With Greed, Prosocial Motivation, and Social Value Orientation.

Variable	*N*	*M*	*SD*	1	2	3
1. Greed (α = .90; ω = .92)	2,367	2.05	0.71			
2. Self-interest (PM; α = .69; ω = .70)	2,367	2.11	0.49	.26***		
3. Self-interest (SVO)	2,367	27.31	9.41	.13***	.23***	
4. Age	2,367	54.06	17.90	–.37***	–.07***	.08***
5. Gender (0 = female, 1 = male)	2,367	0.49	0.50	.14***	.11***	.09***
Economic outcomes						
6. Personal monthly gross income (Euros)	2,244	2380	1988	–.01	.02	–.04
7. Household monthly gross income (Euros)	2,165	4573	2834	.07***	.03	–.08***
Evolutionary outcomes						
8. Number of biological children	2,367	1.47	1.26	–.19***	–.08***	–.00
9. Duration longest relationship (years)	2,118	25.49	17.81	–.26***	–.05*	.05*
10. Number of sexual partners	1,909	4.72	10.46	.08***	.02	.02
Psychological outcomes						
11. Satisfaction with life (α = .91; ω = .93)	2,367	4.91	1.19	–.14***	–.03	–.07**

*Note.* Greed was assessed with the 7-item Dispositional Greed Scale (Seuntjens, Zeelenberg, Van de Ven, & Breugelmans, 2015); Prosocial Motivation (PM) was assessed with the 3-item measure from Eriksson et al. (2020); Social Value Orientation (SVO) was assessed with the 6-item SVO Slider (Murphy et al., 2011). Higher scores indicated more self-interest; Satisfaction with Life was assessed with Diener et al.’s (1985) 5-item Scale. α refers to Cronbach’s alpha, ω refers to McDonald’s omega.

* *p* < .05. **p < .01. ***p < .001.

All three measures also correlated with gender. Males were more greedy and more self-interested. We also observed a negative correlation between greed and age, indicating that the older people were, the less greedy they were. Earlier research also found that males were more greedy than females, and that greed was negatively correlated with age (e.g., Krekels & Pandelaere, 2015; Liu, Sun, Ding, et al., 2019; Seuntjens, Zeelenberg, Van de Ven, & Breugelmans, 2015; Zeelenberg et al., 2020). Age was differently correlated with the two self-interest measures. Age was negatively correlated with self-interest measured as prosocial motivation, suggesting that older people are less self-interested. But age was positively correlated with self-interest measured as SVO, suggesting that older people are more self-interested.

To investigate whether dispositional greed differed from self-interest as measured by the prosocial motivation scale, we conducted a confirmatory factor analysis (CFA), testing whether a unidimensional model (in which one factor would represent greed and self-interest) fitted the data better than a two-factor model (in which greed and the self-interest were represented by separate factors). If dispositional greed is different from self-interest, the two-factor model would result in a better fit than the unidimensional model. Similar to what Seuntjens, Zeelenberg, Van de Ven, and Breugelmans (2015) found for the SVO measure (Van Lange et al., 1997), the two-factor model fit better (had a significantly lower χ^2^) than a unidimensional scale, ∆χ^2^(1) = 1,122.70, *p* < .001, indicating that greed is different from this measure of self-interest.

### Economic Outcomes

Greed correlated positively with household income, but not with personal income.^9^ In contrast to findings by Eriksson et al. (2020), self-interest measured as prosocial motivation did not correlate with personal and household income. Self-interest measured as SVO did correlate negatively with household income, but not with personal income. Thus, at the household level, greedy individuals earn more and self-interested individuals earn less, but at the individual level, no such differences emerge.

### Evolutionary Outcomes

Greed correlated negatively with the number of (biological) children. For self-interest, the findings were mixed; there was a negative correlation between self-interest measured as prosocial motivation and biological children, replicating Eriksson et al. (2020), but there was no a significant correlation for self-interest measured as SVO.

For relationship length, the greedier people were, the shorter their relationships were. For self-interest, we again found different results for the two measures; self-interest measured as prosocial motivation correlated negatively with relationship length, but self-interest measured as SVO correlated positively with relationship length.

Greed correlated positively with the number of sexual partners. Contrary to the predictions, we found no significant correlation between both self-interest measures and the number of sexual partners.

### Psychological Outcomes

Greed correlated negatively with well-being, replicating earlier findings (Krekels & Pandelaere, 2015; Li et al., 2021; Masui et al., 2018; Okulicz-Kozaryn et al., 2021; Seuntjens, Zeelenberg, Van de Ven, & Breugelmans, 2015; Zeelenberg et al., 2020). For the two self-interest measures, the results were mixed. SVO correlated negatively with well-being, while PM and well-being were uncorrelated.

### Partial Correlations

To further differentiate between greed and self-interest, we also looked at the partial correlations of these constructs with the various life outcomes. Results can be found in Table 4. We found that greed and self-interest independently relate to household income and subjective well-being. Furthermore, we found that greed continued to be related to evolutionary outcomes, when controlling for self-interest, but that the effects of self-interest disappeared when controlling for greed.

**Table 4.** Partial Correlations of Greed and Self-Interest Measured as Prosocial Motivation (PM) and Social Value Orientation (SVO) With Economic, Evolutionary, and Psychological Outcomes.

Variable	*N*	Greed		Self-interest (PM)	Self-interest (SVO)
		Controlling for self-interest (PM)	Controlling for self-interest (SVO)	Controlling for greed	Controlling for greed
Personal monthly gross income (Euros)	2244	–.02	–.01	.02	–.03
Household monthly gross income (Euros)	2165	.07**	.08***	.01	–.09***
Number of biological children	2367	–.17***	–.19***	–.03	.02
Duration longest relationship (years)	2118	–.26***	–.27***	.02	.09***
Number of sexual partners	1909	.07**	.07**	.00	.01
Satisfaction with life	2367	–.13***	–.13***	.01	–.05***

*Note.* Greed was assessed with the 7-item Dispositional Greed Scale (Seuntjens, Zeelenberg, Van de Ven, & Breugelmans, 2015); Prosocial Motivation (PM) was assessed with the 3-item measure from Eriksson et al. (2020); Social Value Orientation (SVO) was assessed with the 6-item SVO Slider (Murphy et al., 2011). Higher scores indicated more self-interest. Satisfaction with Life was assessed with Diener et al.’s (1985) 5-item Scale.

** *p* < .01. ***p < .001.

### Exploratory Analyses

We observed significant correlations between our measures for greed and self-interest and gender and age (see Table 3). We did not formulate explicit predictions regarding these correlations; and following the preregistration, we explored the partial correlations between greed and self-interest and life outcomes while controlling for gender and age. Controlling for gender did not affect the significance of any of the relationships between greed and the various life outcomes. Controlling for age did have some effect. The negative relationship between greed and life satisfaction remained significant after controlling for age and gender, *r*(2,367) = –.15 and *p* < .001. The positive relationship between greed and number of sexual partners became nonsignificant, *r*(1,909) = .04 and *p* = .080. For SVO, after controlling for age and gender, there was a significant negative relationship with life satisfaction, *r*(2,367) = –.07 and *p* < .001, and household income, *r*(2,165) = –.07 and *p* < .001. Interestingly, the negative relationship with personal income became significant, *r*(2,244) = –.09 and *p* < .001. For Prosocial Motivation, a significant negative relationship with number of biological children remained, *r*(2367) = -.04 and *p* = .031. The other significant relationships disappeared.

For completeness and to compare our results directly to those reported by Eriksson et al. (2020), we also ran the analyses used in their Study 1 on our data (note that these analyses were not preregistered).^10^ The full procedure and results are reported in Supplemental Appendix 2. The results were the same as the ones from the analyses reported here: replicating Eriksson et al., number of children was positively related to prosocial motivation; not replicating Eriksson et al., neither personal income nor household income were related to prosocial motivation.

To test for measurement models and account for measurement error, we assessed the goodness-of-fit of the measurement models for greed, self-interest (measured as prosocial motivation), and life satisfaction. Note that since self-interest (measured as prosocial motivation) only contains three items, the measurement model is just identified (i.e., it will result in perfect fit), and thus we evaluated its fit in a model with greed, by allowing the two factors capturing self-interest and greed to be correlated. Both the fit for the measurement model including self-interest and greed, χ^2^(34) = 724.49, *p* < .001, comparative fit index (CFI) = .94, Tucker–Lewis index (TLI) = .92, RMSEA = .09, SRMR = .05, and for the measurement model including life satisfaction, χ^2^(5) = 265.24, *p* < .001, CFI = .97, TLI = .94, RMSEA = .15, SRMR = .04 were acceptable. In addition, through structural equation modeling (SEM), we evaluated the relationships between these measures and self-interest (measured as SVO), personal income, household income, number of biological children, relationship length, and number of sexual partners as manifest indicators. All the above-mentioned models were estimated using the lavaan package in *R* (Rosseel, 2012). We used Full Information Maximum Likelihood (FIML) to account for missing data. The corresponding correlations can be found in Table 5. Note that these analyses were not preregistered. The results change somewhat, and the most notable changes are the following. We now find a negative relationship between greed and personal income. For household income, we now find a positive relationship with self-interest (measured as prosocial motivation), and the negative relationship with self-interest (measured as SVO) disappeared. The positive correlation between greed and the number of sexual partners also disappeared (*p* = .077).

**Table 5.** Correlations With Greed, Prosocial Motivation, and Social Value Orientation From the Structural Equation Model With Greed.

Variable	1	2	3
1. Greed			
2. Self-interest (PM)	.27***		
3. Self-interest (SVO)	.14***	.25***	
Economic outcomes			
4. Personal monthly gross income (Euros)	–.06*	–.00	–.00
5. Household monthly gross income (Euros)	.15***	.09**	–.00
Evolutionary outcomes			
6. Number of biological children	–.10***	–.08**	–.03
7. Duration longest relationship (years)	–.20***	.02	.08**
8. Number of sexual partners	.04	.01	.03
Psychological outcomes			
9. Satisfaction with life	–.14***	–.05*	–.08***

*Note.* Prosocial Motivation and Life Satisfaction as Latent Variables. Greed was assessed with the 7-item Dispositional Greed Scale (Seuntjens, Zeelenberg, Van de Ven, & Breugelmans, 2015); Prosocial Motivation (PM) was assessed with the 3-item measure from Eriksson et al. (2020); Social Value Orientation (SVO) was assessed with the 6-item SVO Slider (Murphy et al., 2011). Higher scores indicated more self-interest; Satisfaction with Life was assessed with Diener et al.’s (1985) 5-item Scale.

* *p* < .05. **p < .01. ***p < .001.

## Discussion

What is greed good for? In a representative sample of the Dutch population, we studied relationships between greed and a number of economic, evolutionary, and psychological life outcomes, similar to the approach that Eriksson et al. (2020) recently used to test the possible benefits of self-interest. We examined whether individual differences in dispositional greed (assessed by the Dispositional Greed Scale of Seuntjens, Zeelenberg, Van de Ven, & Breugelmans, 2015) are related to personal and household income (economic outcomes), to number of biological children, sexual partners, and duration of romantic relationships (evolutionary outcomes), and to life satisfaction (psychological outcomes). For comparison, we performed similar analyses for self-interest (assessed via both the Prosocial Motivation scale of Eriksson et al., 2020, and the SVO-slider of Murphy et al., 2011). What did we find?

With the exception of personal income, which we will come back to later, all (preregistered) hypothesized relations with greed were found to be significant: being higher in dispositional greed correlated with having a higher household income and having had more sexual partners, and with having fewer children, shorter lasting romantic relationships and having lower well-being. Importantly, these patterns are different from those of self-interest, where fewer significant relationships were found and where, with the SVO measure, there was a negative correlation with household income and a positive correlation with length of romantic relationships. Greed and self-interest (measured as Prosocial Motivation) where similar in their negative relation with the number of children. The general picture that emerges is that dispositional greed may be good for the purposes of acquisition, but that in a contemporary Western society, in this case, the Netherlands, it confers few other benefits. This largely negative view of greed aligns well with the general condemnation of greed as a sin and with the undesirability of being called a greedy person. However, at closer scrutiny, the results may hint at a more nuanced picture.

To start with economic outcomes, the data show a mixed picture: greedier people did not have a higher personal income than less greedy people, but they did report a higher household income. As was discussed in the introduction, findings on dispositional greed and personal income in previous studies have been mixed (Seuntjens et al., 2016; Seuntjens, Zeelenberg, Van de Ven, & Breugelmans, 2015; Van Muijen & Melse, 2015; Zeelenberg et al., 2020). Interesting in this regard is that in their study among 120.000 Dutch employees, Van Muijen and Melse did report positive relationships for specific occupations, such as sales managers, where greedy individuals earned substantially more than their less-greedy coworkers. This could suggest that the economic benefits of greed are dependent on the specific situation people find themselves in. Unfortunately, our data do not include information on participants’ occupations or the specific branches they were working in. We believe further differentiation along different occupations to be an interesting avenue for future research.

Personal income can come from various sources, such as employment, social benefits, and pensions. Interestingly, exploratory analysis revealed that, among the (self)employed, there was a slight positive relationship between greed and personal income suggesting that greed may indeed be beneficial for personal income in specific situations (i.e., employment). Over all sources of personal income together, however, our data showed a net null effect of personal income (though in the SEM there was a positive correlation).

The positive relationship between greed and household income could go different ways. It might be that greedy individuals contribute to higher household income by, for example, stimulating their partners to work harder, or that greedy individuals select partners that are economically better off, or it could be that there is a third variable, such as greedy individuals taking care of fewer children which contributes to household income because both partners can work more. Context effects may also be important in this regard. Recently, evidence has been found that growing up in more wealthy circumstances is associated with higher greed at a later age (Hoyer, Zeelenberg, & Breugelmans, 2021; Liu, Sun, & Tsydypov, 2019). Thus, it might be that the opportunities that the environment presents breeds higher greed which in turn creates a later preference for environments that are more conducive to greed. Of course, such mechanisms are mere conjecture at the time, but we feel that the question as to when (rather than whether) greed is related to more income is worthy of further attention. It is also interesting that we did not find a relation between self-interest and personal income, while SVO self-interest showed a negative relation with household income, suggesting that there is something specific to greed in this regard.

With regard to evolutionary outcomes, the data suggest that greedy people are more likely to follow an *r*-strategy (MacArthur & Wilson, 1967), having more sexual partners (though in the SEM this relation was not significant) but less long-lasting relationships. In contemporary societies, this may lead to having fewer children, as is evident in our data. However, like the economic outcomes, this effect may be dependent on context. In other social or historical circumstances, an *r*-strategy may actually lead to having increased reproductive opportunities by having more sexual partners, and as a possible consequence, more genetically diverse offspring. From a more psychological perspective, there may also be other reasons why greedy people have fewer children. It could be a deliberate choice but also the result of unsuccessful relational bonding. Interestingly, an exploratory analysis revealed that greedy individuals more often reported having a partner, *r*(2,367) = .05, *p* = .016, but these relationships did not seem to last. In either case, the data suggest that greed transcends mere material goods and acquisitions in that it is related to different ways in which people approach relationships as well. Indeed, this was also suggested by Hoyer (2022), who found that, among other things, greedy individuals objectify their friends more and feel less close to them.

With regard to psychological outcome, the data are quite clear and very much in line with previous research (Krekels & Pandelaere, 2015; Li et al., 2021; Masui et al., 2018; Seuntjens, Zeelenberg, Van de Ven, & Breugelmans, 2015; Zeelenberg et al., 2020); higher dispositional greed was related to a lower satisfaction-with-life. This could be an intrinsic property of greed: the constant dissatisfaction of never having enough and the endless pursuit of more which are core characteristics of greed may by necessity imply lower life satisfaction in general. The relationship could also be more indirect, with greedy people being less satisfied with life due to the fact that, for example, their relationships are shorter lasting or their families are smaller. Having good social relationships is crucial to well-being (e.g., Amati et al., 2018), even more so than having a good income (Powdthavee, 2008).

A secondary goal of this study was to compare greed with self-interest. The reasons for including this comparison were two-fold. First, self-interest and greed are clearly related constructs, both theoretically and empirically. Second, the design of our study was inspired by the study of Eriksson et al. (2020). For a complete comparison, we not only used the Prosocial Motivation Scale that was used by Eriksson et al. to measure self-interest, but also the more commonly used SVO-slider (Murphy et al., 2011). Both measures gave slightly different results.

When comparing the bivariate correlational results, it is notable that greed and self-interest share many of the negative relationships, although self-interest shows overall fewer significant relationships. One salient difference is the relationship with household income, which is positively related to greed but negatively to self-interest. A second difference is the relationship with duration of the longest romantic relationship. Greed was related to shorter romantic relationships, while for self-interest the effects depended on the scale: self-interest as measured by Prosocial Motivation was negatively correlated with relationship length, but self-interest as measured by SVO was positively correlated. This makes the interpretation of the results in relation to greed complicated. A third difference is that greed was positively related to the number of sexual partners whereas there was no significant relationship with self-interest. Thus, being greedy appears to be somewhat more advantageous than being self-interested, both economically and evolutionarily.

Because greed and self-interest were correlated, we also looked at partial correlations. Here, unique effects of greed and self-interest remain, albeit only for the SVO measure. When it comes to greedy and self-interested individuals having fewer children, partial correlations suggests that this effect may better be explained by greed than by self-interest. The same holds for the negative correlation with relationship length of the prosocial motivation measure. The positive correlation of the SVO measure with relationship length remained significant after controlling for greed. Also, the negative correlation with life satisfaction remained significant for both greed and SVO-self-interest, suggesting that being greedy and being self-interested makes you unhappy in their own way.

Taken together, these results clearly show the usefulness of distinguishing between greed and self-interest when it comes to studying economic, evolutionary, and psychological outcomes. All in all, greed appears to have positive and negative relationships with life outcomes, whereas self-interest tends to be negative across the board for the outcomes that we examined.

To account for measurement error, we used SEM to further explore the relationship between greed and self-interest as latent variables and the economic, evolutionary, and psychological outcomes as manifest indicators. The results changed somewhat, indicating that we should interpret the results with caution. The most notable changes are the following. Using SEM, we found a negative relationship between greed and personal income. For household income, SEM revealed a positive relationship with self-interest (measured as prosocial motivation), and the negative relationship with self-interest (measured as SVO) disappeared. The positive correlation between greed and the number of sexual partners disappeared (*p* = .077) in SEM.

Like any study using correlational, cross-sectional panel data, there are limitations to this study. Ideally, a future, longitudinal study should investigate the underlying mechanism of differences in greed in socioeconomic success over the years. The results obtained for income already suggest that there might be differences over the course of people’s lives. Furthermore, future research could investigate why the greedy have lowered evolutionary outcomes by examining the mating practices of the greedy. Finally, future research should investigate why greedier individuals feel less satisfied with life, in order to design interventions to increase their mental well-being and reduce possible severe side effects such as depression.

A second limitation that would warrant more research is the observation of a relatively strong correlation between greed and age in our data. When we explored the effect of age as a control variable many relationships between greed (as well as self-interest) and life outcomes were no longer significant. Given the limited literature on such effects, it is hard to provide a strong interpretation as to what this means. Both Liu, Sun, and Tsydypov (2019) and Hoyer, Zeelenberg, and Breugelmans (2021) speculated that a relationship between age and greed might be curvilinear, following an inverted *U*-shape. This would mean that greed reaches a maximum in early adulthood. In favor of such a relationship are findings of a positive correlation between greed and age with adolescent samples (Liu, Sun, & Tsydypov, 2019; Seuntjens et al., 2016), and findings of negative relationships with adult samples (Liu, Sun, Ding, et al., 2019; Seuntjens, Zeelenberg, Van de Ven, & Breugelmans, 2015). With regard to self-interest and age, we found somewhat mixed evidence: Age correlated positively with self-interest measured with SVO but negatively with self-interest measured with Prosocial Motivation. The literature seems to be more in line with the latter, suggesting that people become more prosocial later in life (e.g., Matsumoto et al., 2016). Van Lange et al. (1997) refer to this phenomenon as the *prosocial-growth hypothesis.* It would appear to be worthwhile to further investigate the relationships among greed, self-interest and age, especially from a developmental, longitudinal perspective.

In this research, we used two existing measures of self-interest: the inverse of prosocial motivation (Eriksson et al., 2020) and SVO (Murphy et al., 2011). Both measures correlated, but not highly, and correlations with the different life outcomes differed somewhat between measures. This raises questions about the convergent validity of both self-interest measures. Eriksson et al. analyzed a large number of existing data sets, so in their search for indicators of self-interest, they were bound by what was available. The prosocial motivation scale was used in Study 1, but in other studies, they employed different indexes. In retrospect, we believe that the operationalization as self-interest as the inverse of prosocial motivation might be criticized from a psychological perspective. In organizational research, people have argued for treating self-interested and other-interested orientations as distinct dispositions (e.g., Meglino & Korsgaard, 2004). Research of Gerbasi and Prentice (2013) shows that indeed self-interest and other-interest were moderately positively correlated, rather than negatively correlated. For this reason, we included the SVO measure of Murphy et al. (2011), which is based on decomposed games, as a more traditional measure of a continuum between prosociality and self-interest. This measure is closer to how self-interest is usually assessed in psychological research. However, because our study was not designed to distinguish between different indicators of self-interest, we refrain from speculating on the difference between the two measures in too much detail. Most important for this article is that the patterns of both self-interest measures were distinct from that of greed.

A question that could be asked is to what extent the relationships we found for greed are unique to this construct or whether they could be explained by other constructs. Previous research by Seuntjens, Zeelenberg, Van de Ven, and Breugelmans (2015) revealed four constructs that most strongly correlate with greed: in decreasing order materialism, envy, maximization, and self-interest. The latter construct was the comparison standard in this article but could the other constructs explain the effects of greed? We believe that this is not plausible. First, in a multistudy prototype analysis, Seuntjens, Zeelenberg, Breugelmans, and Van de Ven (2015) found that although these constructs were mentioned, they did not belong to the core of features of greed. It is this core that is assessed by the DGS that we used in this study. In addition, Seuntjens, Zeelenberg, Van de Ven, and Breugelmans (2015) extensively mapped the nomological network of the DGS and the other constructs. Greed emerged as being clearly distinct. Furthermore, there are theoretical reasons why the other constructs cannot explain the full pattern that we found for greed. For example, the research by Crusius and Lange (2021) suggests that greed predicts envy, and not the other way around. Likewise, while materialism might well be related to income, the relationship with number of children, relationships and number of sexual partners is not at all evident. Finally, maximization might be related to more sexual encounters but should rather relate to having more rather than fewer children. Thus, we are somewhat confident that the patterns we found for greed are unique to greed in comparison to related constructs.

Another question that might arise is whether certain conditions that might be unique to a particular country or culture has a significant effect on the outcome variables in our study. Although we do not have direct evidence for cross-cultural equivalence, we have quite a bit of evidence for the cross-cultural validity and invariance of structural relations for the Dispositional Greed Scale from Seuntjens, Zeelenberg, Van de Ven, and Breugelmans (2015). This scale has been applied in (and validated for use in) various countries from different continents. Furthermore, many effects of greed are structurally the same between these samples, for instance positive associations between the greed and envy, psychological entitlement, materialism, and impulsive buying behavior, and negatively associations between greed and self-control, self-esteem, and life satisfaction. As a further case in point, recent evidence for the luxury hypothesis (that growing up wealthy is related to higher levels of adult greed) as found in a Chinese sample (Liu, Sun, & Tsydypov, 2019) has been replicated in Dutch and American samples (Hoyer, Zeelenberg, & Breugelmans, 2021). Of course, none of this is direct evidence for cultural invariance, and we cannot exclude that there are global conditions that would lead to different relations. However, given the extant evidence, we believe that we can be reasonably confident that our findings are not limited to the Netherlands as a country or a culture.

Let us return to the question that motivated the current research, is there anything good about greed? Despite the clear condemnation of greed in philosophical, religious, and popular writings, our results show that greed is (somewhat) beneficial for economic outcomes (supporting claims put forward by some economists). However, our results also show that greed is mixed for evolutionary outcomes and unfavorable for psychological outcomes. A secondary goal of this study was to disentangle the relationship between greed and self-interest. On the basis of the current findings, we can say that greed and self-interest differ in their relation to economic outcomes and are mostly similar in their relation to evolutionary outcomes (with greed being somewhat more advantageous) and well-being. In short, greed may be good for income but bad for happiness.

## Supplemental Material

sj-docx-1-psp-10.1177_01461672221140355 – Supplemental material for Greed: What Is It Good for? Supplemental material, sj-docx-1-psp-10.1177_01461672221140355 for Greed: What Is It Good for? by Karlijn Hoyer, Marcel Zeelenberg and Seger M. Breugelmans in Personality and Social Psychology Bulletin

## Notes

1. This is an interesting observation because some other research on self-interest and other-interest found that these constructs are not necessary opposites and even correlate slightly positively (Gerbasi & Prentice, 2013).

2. The relationship that Eriksson et al. (2020) reported was not strictly linear, which we address in the results section.

3. The preregistration includes two additional variables: Childhood Socioeconomic Status (CSES; Griskevicius et al., 2011) and number of siblings, which were used for another project.

4. Raw data and the corresponding code book can be freely downloaded at https://www.dataarchive.lissdata.nl/study_units/view/889.

5. For more information visit www.lissdata.nl.

6. We found no order effects on the DGS (*p* = .351), *M*_start_ = 2.03, and *M*_end_ = 2.06.

7. Personal income also includes income from sources other than employment, such as social securities and pensions. In our sample, 1,083 respondents reported to be either employed or self-employed. Notably, greedy individuals are more likely to be employed than their nongreedy counterparts, *r*(2,367) = .13 with *p* < .001.

8. The imputation procedure can be found on https://www.dataarchive.lissdata.nl/hosted_files/download/1579.

9. Notably, for participants who receive income from either employment or self-employment (and excluding the 12 participants who reported zero income despite being employed), we found a slight positive relationship between greed and personal income, *r*(1,012) =.06, *p* = .048.

10. We thank Irina Vartanova for providing us with the code for their analyses, so that we could replicate it with our data.
