# Supplementary material for: Greed: What Is It Good for?
Source: Pers Soc Psychol Bull. 2022 Dec 28;50(4):597–612. doi: 10.1177/01461672221140355 (PMC10903135; doi:10.1177/01461672221140355)
Supplement: sj-docx-1-psp-10.1177_01461672221140355 – Supplemental material for Greed: What Is It Good for? [file sj-docx-1-psp-10.1177_01461672221140355.docx]

**APPENDIX 1: Full correlation table**

Table A.2

*Correlations between all variables*

| Variable | 1 | 2 | 3 | 4 | 5 | 6 | 7 | 8 | 9 | 10 |
| --- | --- | --- | --- | --- | --- | --- | --- | --- | --- | --- |
| 1. Greed (α = .90; ω = .92) |  |  |  |  |  |  |  |  |  |  |
| 2. Self-interest (PM; α = .69; ω = .70) | .26** |  |  |  |  |  |  |  |  |  |
| 3.Self-interest (SVO) | -.13** | .23*** |  |  |  |  |  |  |  |  |
| 4. Age | -.37** | -.07*** | -.08** |  |  |  |  |  |  |  |
| 5. Gender (0 = female, 1 = male) | .14** | .11*** | -.09** | .05* |  |  |  |  |  |  |
| Economic outcomes |  |  |  |  |  |  |  |  |  |  |
| 6. Personal monthly gross income (Euros) | -.01 | .02 | .04 | .13*** | .34*** |  |  |  |  |  |
| 7. Household monthly gross income (Euros) | .07** | .03 | .08** | -.18*** | .05* | .54*** |  |  |  |  |
| Evolutionary outcomes |  |  |  |  |  |  |  |  |  |  |
| 8. Number of biological children | -.19** | -.08*** | -.00 | .44*** | -.03 | .08*** | .07** |  |  |  |
| 9. Duration of longest relationship (years) | -.26** | -.05* | -.05* | .74*** | .02 | .02 | -.04 | .51*** |  |  |
| 10. Number of sexual partners | .08** | .02 | -.02 | -.06* | .11*** | .07** | -.05 | -.12*** | -.19*** |  |
| Psychological outcomes |  |  |  |  |  |  |  |  |  |  |
| 11. Satisfaction with Life (α = .91; ω = .93) | -.14** | -.03 | .07** | .01 | -.00 | .12*** | .19*** | .09*** | .11*** | -.04 |

*Note.* Greed was assessed with the 7-item Dispositional Greed Scale (Seuntjens et al., 2015b); Prosocial Motivation (PM) was assessed with the 3-item measure from Eriksson et al. 2020; Social Value Orientation (SVO) was assessed with the 6-item SVO Slider (Murphy et al., 2011). Higher scores indicated a more proself orientation; Satisfaction with Life was assessed with Diener et al.’s (1985) 5-item Scale. α refers to Cronbach’s alpha, ω refers to McDonald’s omega.

* *p* < .05. ** *p* < .01. *** *p* < .001.

**APPENDIX 2: Current Results Compared to Eriksson et al. (2020)**

In order to compare our results directly to those reported by Eriksson et al. (2020), we also ran their analyses of the General Social Survey (GSS) data as presented in their Study 1.^[[1]](#footnote-1)^ To this avail prosocial motivation was coded such that higher scores indicated less selfishness and more prosocial motivation. As in Eriksson et al. the prosocial motivation measure was (somewhat) negatively skewed, so this measure was bottom-coded at the lowest percentile to reduce sensitivity to outliers. The measure was then normalized to range between 0 and 1. The final measure had a median of .57, a mean of .52 and a *SD* .21 (comparable to Eriksson et al.: *Mdn* = .62, *M* = .56, and *SD* = .47). The correlation between greed and the resulting prosocial motivation measure was *r*(2367) = -.25, indicating that higher greed scores accompany higher levels of selfishness. The correlation between SVO and the resulting prosocial motivation measure was *r*(2367) = -.22, indicating that higher selfishness scores on the SVO measure correspond to higher levels of selfishness on the prosocial motivation measure.

In the income models, respondents who did not earn any income were excluded and income variables were log transformed. All models included controls for gender and age. In the models with number of children as dependent variable, the age effect was controlled for with penalized cubic spines in generalized additive models. The analyses of number of children used Poisson models, and the analyses of income used linear models. Quadratic models were fitted to account for a possibly nonlinear relation between being prosocial and income/number of children. We selected the best fitting model for number of children, personal income and household income according to the Bayesian information criterion (Schwarz, 1978). Below we present the results of the best fitting models, which in all cases turned out to be the linear models.

Replicating Eriksson et al. (2020), number of children was positively related to prosocial motivation. The unstandardized regression coefficient was *B* = .020, *t*(2367) = 2.39, *p* = .017. Not replicating Eriksson et al., neither personal income nor household income were related to prosocial motivation. The unstandardized regression coefficient for personal income was *B* = -0.05, *t*(2013) = -0.61, *p* = .543; The unstandardized regression coefficient for household income was *B* = -0.08, *t*(2144) = -1.07, *p* = .286.

1. We thank Irina Vartanova for providing us with the code for their analyses, so that we could replicate it with our data. [↑](#footnote-ref-1)
